# A mobile target

**DOI:** 10.7554/eLife.86697

**Published:** 2023-03-08

**Authors:** Carolina Oliveira de Santana, Pieter Spealman, Gabriel G Perron

**Affiliations:** 1 Center for Environment Sciences and Humanities, Bard College Annandale-on-Hudson United States; 2 Center for Genomics and Systems Biology, New York University New York United States

**Keywords:** antibiotic resistance, aminoglycosides, ecology, globalization, antibiotic consumption, Other

## Abstract

The global spread of antibiotic resistance could be due to a number of factors, and not just the overuse of antibiotics in agriculture and medicine as previously thought.

**Related research article** Pradier L, Bedhomme S. 2023. Ecology, more than antibiotics consumption, is the major predictor for the global distribution of aminoglycoside-modifying enzymes. *eLife* **12**:e77015. doi: 10.7554/eLife.77015.

Antibiotic resistance – the ability of bacteria to survive even the strongest clinical treatments – continues to be a major public health concern around the world (Antimicrobial Resistance Collaborators, 2022). The overuse of antibiotics in medicine and agriculture is often thought to be the driving force behind the emergence and spread of bacteria that are resistant to antibiotics (Cohen, 1992; Levy and Marshall, 2004). Overuse can certainly explain the selection for bacteria with genes for antibiotic resistance in environments that are heavily impacted by human activity (Gaze et al., 2013; Kraemer et al., 2019), but it cannot explain the widespread distribution of genes for resistance to clinically relevant antibiotics well away from hospitals and farms. Indeed, such genes have even been found in environments as remote as the Arctic permafrost (D’Costa et al., 2011; Perron et al., 2015) and Antarctica (Hwengwere et al., 2022; Marcoleta et al., 2022).

Now, in eLife, Léa Pradier and Stéphanie Bedhomme of the University of Montpellier report the results of a study that sheds light on this matter (Pradier and Bedhomme, 2023). Focusing on resistance against aminoglycoside, a widely used family of antibiotics that includes streptomycin (Davies and Wright, 1997), the researchers conducted one of the largest surveys of antibiotic resistance genes to date. They analyzed more than 160,000 bacterial genomes collected from all over the globe, focusing on 27 clusters of genes that code for aminoglycoside-modifying enzymes (also known as AME genes). The bacteria were sampled between 1885–2019, although most were sampled recently, with the first example of a bacterium with an AME gene dating from 1905. In addition to the location and date of sampling, the study also considered the number of antibiotics consumed in each country, commercial trade routes, and human migration. Finally, the samples came from eleven different biomes, representing a range of environments where antibiotic resistance can be found: clinical environments (like hospitals), human habitats, domestic animals, farms, agrosystems, wild plants and animals, freshwater, seawater, sludge and waste, soil, and humans.

The researchers found that the prevalence of genes for aminoglycoside resistance increased between the 1940s and the 1980s, likely following an increase in the use of antibiotics after the discovery of streptomycin in 1943 (Schatz et al., 1944), and then remained at a prevalence of around 30%, despite an overall decrease in consumption. Crucially, they also discovered that around 40% of the resistance genes were potentially mobile, which means they can be easily exchanged between bacteria.

Pradier and Bedhomme also found that antibiotic-resistant bacteria are present in most biomes, and not just in hospitals and farms. Moreover, they found that the prevalence of aminoglycoside resistance genes varied more from biome to biome than it did with human geography or with the quantity of antibiotics used (Figure 1). This means that the antibiotic resistance found in humans in one country is more likely to be related to the antibiotic resistance found in humans in a distant country than it is to the antibiotic resistance found in the soil or animals nearby. Moreover, they also discovered that biomes such as soil and wastewater likely play a key role in spreading the genes for antibiotic resistance across different biomes.

**Figure 1.**
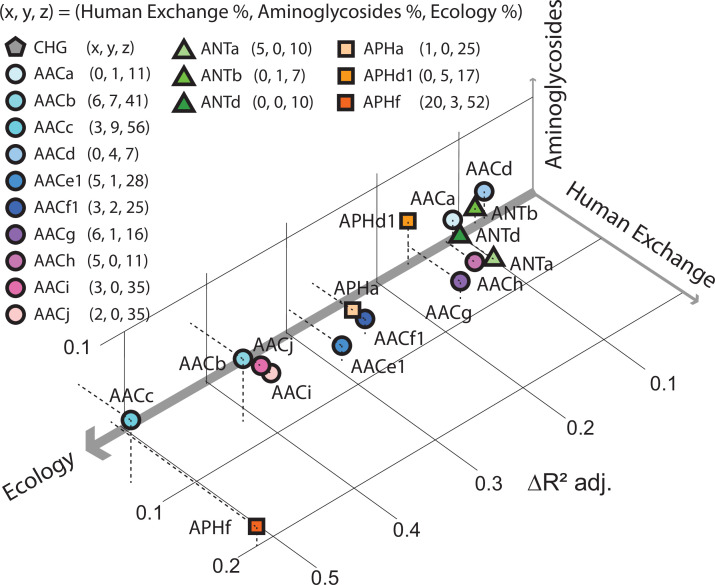
Factors influencing the spread of resistance against aminoglycoside antibiotics. Pradier and Bedhomme analyzed the variables that influence the prevalence of genes that code for aminoglycoside-modifying enzymes (AME genes) in a sample of 160,000 bacterial genomes collected from all over the globe. The samples were classified as belonging to one of eleven biomes (see main text). It was found that ecology (that is, which of the 11 biomes the sample was collected from) was the biggest influence, followed by human exchange (immigration and material import/export) and aminoglycoside consumption. This scatter plot for samples collected in Europe between 1997 and 2018, which is derived from Figure 5 of Pradier and Bedhomme, 2023, shows how the prevalence of 16 clusters of these genes depends on these three variables, with the thickness of each axis representing how influential that variable was overall (ecology, 80%; human exchange, 13%; aminoglycoside consumption, 7%; interactions between the variables are not included). The clusters of genes represented by circles code for N-acetyltransferases; triangles represent nucleotidyltransferases, and squares represent phosphotransferases.

These findings raise important questions about the mechanisms underlying the spread of antibiotic resistance. What factors promote the spread of antibiotic resistance in environments not impacted by human activities? Can we extrapolate these results from the aminoglycosides to all other classes of antibiotics? Is it possible that antibiotic resistance results from interactions with local microbial communities more than exposure to commercial antibiotics? Do the genes for antibiotic resistance spread in the pathogenic bacteria responsible for human and animal infections in the same way as they spread in non-pathogenic bacteria? Given the extent of the selective pressure exerted by human pollution, what limits the spread of antibiotic-resistance genes between biomes, especially given the large proportion of genes on mobile elements?

It may well be that consumption still has a paramount role when it comes to resistance to the antibiotics that are used to treat infection, especially in humans and clinical biomes. Nevertheless, it is clear that we need to pay more attention to the role of the environment when formulating plans to combat antibiotic resistance on a global scale.
